# Prevalence, Determinants, and Barriers to Reproductive Health Decision‐Making Autonomy Among Married Women in Rural Parts of Seden Sodo District, Southwest Ethiopia: A Mixed‐Methods Study

**DOI:** 10.1002/hsr2.71791

**Published:** 2026-01-26

**Authors:** Shimelis Gobena, Seifadin Ahmed Shallo, Gemechu Gelan Bekele, Eden Girmaye Tefera, Gizachew Abdissa Bulto

**Affiliations:** ^1^ Southwest Shewa Zone Health Office, Oromia Regional Health Bureau Wolliso Ethiopia; ^2^ Department of Public Health College of Medicine and Health Sciences Arsi University Asella Ethiopia; ^3^ Department of Midwifery College of Health Sciences Madda Walabu University Shashamane Ethiopia; ^4^ Department of Midwifery College of Medicine and Health Sciences Ambo University Ambo Ethiopia

**Keywords:** autonomy, decision‐making, Ethiopia, reproductive health service, Seden Sodo

## Abstract

**Background and Aim:**

In rural Ethiopia, where patriarchal norms prevail, women's limited decision‐making autonomy significantly restricts their reproductive health (RH) service utilization. However, scarcity of data persists. Hence, this study assesses the prevalence, determinants, and barriers to RH decision‐making autonomy among married women in rural parts of Seden Sodo district, Southwest Ethiopia.

**Methods:**

A community‐based mixed‐methods study was conducted from December 2022 to January 2023 among 594 systematically selected married women in the Seden Sodo district, Southwest Ethiopia. The quantitative data were collected using structured interviewer‐administered questionnaires and analyzed by SPSS version 27. Women's decision‐making autonomy was assessed across four key reproductive health areas including family planning, antenatal care, place of delivery, and postnatal care using a weighted scoring system: 2 for sole decision‐maker, 1 for joint decisions, and 0 if the husband decided alone. Women who scored above the mean were considered autonomous. Sixteen key informants participated in qualitative interviews analysed thematically to explore contextual barriers with triangulation of the findings.

**Results:**

Only 53.2% (95% CI: 49–57) of women demonstrated autonomous RH decision‐making. Merchant occupation (AOR = 6.88, 95% CI: 3.12–15.14), age at marriage age > 18 years (AOR = 5.49, 95% CI: 3.20–9.43), husband's education (AOR = 3.51, 95% CI: 2.10–5.90), favourable RH perceptions (AOR = 2.08, 95% CI:1.05–4.12) and women's education (AOR = 0.47, 95% CI: 0.30–0.76) were significantly associated with RH decision‐making autonomy. Qualitative analysis revealed three key barriers: entrenched male dominance in health decisions, female economic marginalization, and mobility restrictions impeding service access.

**Conclusion and Recommendation:**

While approximately half of rural women demonstrate some RH decision‐making autonomy, significant barriers persist. Therefore, programs promoting women's economic participation, challenging patriarchal norms, and engaging male partners are critical to enhancing autonomous RH decisions and improving maternal health outcomes.

**Abbreviations**
AOR: Adjusted Odds Ratio
COR: Crude Odds Ratio
RH: Reproductive Health

## 1 Background

Reproductive health (RH) is a crucial to women's overall well‐being, encompassing pregnancy, childbirth, family planning, and access to essential health services [1]. Women's capacity to access and utilise RH treatments is heavily influenced by their decision‐making autonomy, the ability to make independent choices regarding their health, mobility, finances, and children's well‐being without external approval [2, 3].

Although progress towards gender equality has gradually enhanced some women's decision‐making autonomy, reproductive and sexual autonomy are still severely limited [4, 5]. For instance, research from 57 countries revealed that only 55% of married women aged 15–49 exercise autonomy in reproductive health service utilization [6]. Similarly, the result of one systematic review and meta‐analysis conducted in low‐ and middle‐income countries showed that women's decision‐making autonomy on maternal health services is 55.2% [7].

In recent years, Ethiopia has made significant improvement in RH services utilization, with antenatal care coverage increasing from 62% in 2016% to 74% in 2019, and institutional delivery rates rising from 26% to 48% over the same period [8]. Despite these advances, women's RH decision‐making autonomy remains a significant challenge [9, 10]. Women's RH decision‐making autonomy is severely compromised in Ethiopia, where more than 80% of Ethiopian women live in rural areas where patriarchal norms prevail, resulting in women being socially positioned as subordinates to their husbands [11, 12]. Only 11%–18% of women made decisions concerning their own and their newborn health services alone, while 66%–68% made decisions alongside their spouse or partner [9].

Individual studies in various Ethiopian regions also illustrate varying prevalence of RH decision making autonomy, ranging from 41% [13] to 66% [14]. Moreover, different sociodemographic factors, cultural norms, spousal communication, social supports, and access to information influence women's autonomy [15, 16, 17, 18, 19, 20, 21].

Despite various international efforts initiated to improve maternal health, Ethiopia has one of the world's highest maternal mortality rates, estimated at 412 per 100,000 live births [22]. This greatly exceeds the worldwide target of reducing maternal mortality to less than 70 deaths per 100,000 live births by 2030 [6].

Reducing levels of maternal mortality and morbidity depends on increasing the use of reproductive and maternal health services [7]. Low autonomy restricts RH services utilization, adversely impacting maternal health outcomes and perpetuating gender inequities [23, 24].

The empowerment of women to make autonomous decisions about reproductive health is critical to achieving SDG target 5.6, which seeks universal access to sexual and reproductive health and reproductive rights by 2030 [25]. According to UNFPA's global database on SDG indicator 5.6.1, many low‐ and middle‐income countries, including Ethiopia, face significant challenges in ensuring women's independent decision‐making on RH matters [26].

Existing evidence gaps persist regarding women's RH decision‐making autonomy in Ethiopia's rural settings. The majority of previously conducted studies in Ethiopia also solely used a quantitative technique to assess women's RH decision‐making autonomy. For a better understanding of this problem, this study aimed to assess the prevalence, determinants, and barriers to RH decision‐making autonomy among married women in rural parts of Seden Sodo district, Southwest Ethiopia, using both quantitative and qualitative methods.

## 2 Methods

### 2.1 Study Design, Area, and Period

A community‐based mixed‐methods study was conducted from December 20, 2022, to January 20, 2023, in the Seden Sodo district, Southwest Ethiopia. The quantitative component of the study utilized a cross‐sectional design featuring interviewers administered questionnaires among systematically selected married women of reproductive age. The qualitative component involved key informant interviews with community leaders and healthcare providers to identify barriers affecting women's RH decision‐making autonomy. Seden Sodo is predominantly rural, comprising one urban and 23 rural kebeles, with a population of approximately 112,656, including nearly 21,000 women of reproductive age. The district is located 107 km southwest of Addis Ababa, the capital city of Ethiopia. There are four health centers, 23 health posts, and nine private clinics in the district.

### 2.2 Population

All married women of the reproductive age group who were residing in the Seden Sodo district were the source population, while randomly selected married women of reproductive age who lived in the selected kebeles of the Seden Sodo district at the time of the study and had resided there for at least 6 months were the study population for the quantitative study. Moreover, purposively selected representatives of the communities, like representatives from women and child affairs office, health extension workers, health professionals working on RH services, and community health workers, were the study population for the qualitative study.

### 2.3 Sample Size Determination

Sample size was determined based on the single population proportion formula considering the proportion of married women who have decision‐making power over RH services utilization, which was 40.3%, 95% confidence level, 5% marginal error, a design effect of 1.5 (to account for the potential clustering effect due to the multi‐stage sampling technique), and a 10% non‐response rate. The final sample size was found to be 610.

For the qualitative data, sixteen key informants' interviews were undertaken to assess the barriers to women's decision‐making autonomy on selected RH services utilization.

### 2.4 Sampling Technique and Procedure

Seden Sodo district was purposefully chosen as the study site because it is one of predominantly rural settings in Southwest Ethiopia. Eight kebeles were randomly selected. The sample was proportionally allocated to the selected kebeles based on their population size. Married women of reproductive age with at least one child were listed from health post records to create a sampling frame. Systematic random sampling was employed to select participants. In cases of non‐availability, interviewers re‐visited the household on at least three occasions before excluding them as non‐respondent.

Key informants for the qualitative study were purposively selected based on their knowledge and roles related to reproductive health. Participants were drawn from Women and Children Affairs Office, health extension workers, and RH service providing health professionals. Sampling proceeded until thematic saturation was reached and 16 in‐depth interviews were conducted.

### 2.5 Data Collection Tools and Procedure

Data collection instruments for both qualitative and quantitative studies were developed after thoroughly revising related literatures and adapting questionnaires used in other related studies by considering local conditions and study objectives [11, 14, 27]. The quantitative tool was originally developed in English, then translated into Afan Oromo (the local language of Seden Sodo district, Oromia region), and back‐translated to English for consistency. The Afan Oromo version was used for data collection.

The quantitative tool included 45 items covering socio‐demographics, reproductive history, RH knowledge and perceptions, decision‐making autonomy, and socioeconomic status. The portion assessing women's perceptions of reproductive health services employed a five‐point Likert scale ranging from strongly disagree to strongly agree. The section on women's RH decision‐making autonomy featured items classified as self, jointly with husband, and only husband only, to indicate who had the final say in using services like family planning, prenatal care, skilled delivery, and postnatal care. The qualitative component employed semi‐structured interviews with key informants to explore sociocultural barriers to RH autonomy. Each session lasted between 30 and 45 min, guided by open‐ended questions and probes (Supplemental file 1).

## 3 Measurements

### 3.1 Women's Decision‐Making Autonomy on RH Services Utilization

RH decision‐making autonomy was assessed with four core questions on who made the final decision for modern family planning, antenatal care, place of delivery, and postnatal care. This approach closely follows the practice used in large international and Ethiopian surveys, such as the Demographic and Health Surveys [15]. The responses were scored as follows: 2 if the woman was the sole decider, 1 if the decision involved joint decision‐making with the husband/partner, and 0 if the husband was the sole decision‐maker. A composite score was computed and dichotomized at the mean, with scores above indicating autonomy and below indicating lack thereof [2, 28].

### 3.2 Women's Awareness of Selected RH Services

Awareness was assessed through nine questions on knowledge of modern family planning, antenatal care, skilled delivery, and postnatal services. Responses were coded dichotomously (1 = knowledgeable/familiar, 0 = not). Women scoring 75% or higher ( ≥ 7) were classified as having good awareness, while those below were considered to have poor awareness [28].

### 3.3 Women's Perception Toward Selected RH Services

It was assessed using four statements about RH services, including contraception use, antenatal care, skilled delivery, and postnatal care. Participants rated agreement on a five‐point Likert scale from strongly disagree [1] to strongly agree [5]. Total scores were summed and dichotomized at the mean, with scores above reflecting favourable perceptions and those below indicating unfavourable perceptions [14].

### 3.4 Household Wealth Index

To construct the wealth index as a proxy⁠ for socioeconomic status, we included household assets⁠ and characteristics such as ownership‍ of durable goods⁠ (‌e.g., radio, television, bicycle‌), housing conditions (e.g., ‌ type of flooring, roofing material), water supply, sanitation facilities, and livestock ownership. These variables were entered into a principal component analysis from which‌ factor scores were generated to categorize households into wealth tertiles (poor, middle, rich) [29].

### 3.5 Data Quality Control

Before the actual data collection, the quantitative questionnaire was pretested on 30 randomly selected married women of reproductive age who have at least one child in Illo, an unselected kebele of the district. A 1‐day training was given to the data collectors and supervisors on the objective, confidentiality of information, respondent rights, and techniques of interviewing. Finally, the completeness and consistency of the collected data were reviewed and checked every day by supervisors and investigators. Discussions were made with the interviewers at the end of the day and in the morning; corrective actions were taken promptly to minimize errors committed during the interview.

### 3.6 Data Processing and Analysis

The collected quantitative data was cleaned, coded, and entered into Epidata version 4.6, then exported to SPSS v27 for analysis. Descriptive statistics summarized sample characteristics. Binary logistic regressions identified factors associated with RH decision‐making autonomy, with variables significant at *p* < 0.25 in bivariate analysis entered into multivariable models. Significance was set at *p* < 0.05. The goodness of fit of the final model was checked using the Hosmer‐Lemeshow test at a *p*‐value > 0.05. Multicollinearity among independent variables was assessed using the Variance Inflation Factor (VIF), with a common threshold of VIF ≥ 10 indicating problematic multicollinearity [30]. In our analysis, all variables had VIF values below 3, indicating no significant multicollinearity.

The qualitative data collected through key informant interviews were transcribed verbatim in Afan Oromo and then translated into English by bilingual professionals. Accuracy was ensured via cross‐checking by a second translator and investigators. Data were analysed manually following Braun and Clarke's thematic analysis framework [31]. The coding and theme development were performed independently by two investigators to enhance credibility, with discrepancies resolved through discussion.

### 3.7 Ethical Consideration

The study was conducted in accordance with the Declaration of Helsinki. Ethical approval was obtained from the research review committee of the College of Medicine and Health Sciences, Ambo University, with reference number AU/CPG/093/2015. Official letters of support were obtained from the Seden Sodo district health bureau and the selected kebeles in which the actual data collection was undertaken. Written informed consent was taken from all study participants after a clear description of the objectives of the study and its procedures by the data collectors before data collection. Confidentiality was maintained, and data were anonymized to prevent identification.

## 4 Results

### 4.1 Socio‐Demographic Characteristics of Respondents

From the total of 610 eligible women, 562 women responded fully to the study, yielding a response rate of 92.1%. The mean age of the study participants was 32 years, with a standard deviation (SD) of ±7.82. A large proportion of the respondents (64.1%) were Orthodox. More than half (55.7%) had no formal education. The majority (85.4%) of the women were housewives (Table 1).

**Table 1:** Socio‐demographic characteristics of married rural women of reproductive age in rural parts of Seden Sodo district, Southeast Ethiopia, 2023 (*N* = 562).

Variables	Categories	Frequency	Percentage
Age category (in years)	18–24	128	22.8
	25–34	162	28.8
	35–44	255	45.4
	45–49	17	3
Religion	Orthodox	360	64.1
	Muslim	105	18.7
	Protestant	97	17.3
Women educational status	No formal education	313	55.7
	Formal education	249	44.3
Women occupation	Housewives	480	85.4
	Merchants	82	14.6
Husbands' education	No formal education	384	68.5
	Formal education	177	31.5
Husbands' occupation	Farmer	429	76.3
	Merchant	81	14.4
	Daily labour	52	9.3
Wealth status	Poor	96	37.1
	Middle	77	29.7
	Rich	86	33.2

### 4.2 Reproductive Characteristics of Study Participants

The mean age at first marriage of study participants was 17.98 years, with SD of ± 1.4 years. The mean duration since marriage of study participants was 13.7 years, with SD of ± 7.83 years. Among study participants, the average number of children was 3.57. The majority of study participants (66.8%) had parity of ≤ 4. The mean age of birth interval of the last children among study participants was 2.44 years, with SD of 1.95 years.

### 4.3 Perception & Awareness of the Study Participants on Selected RH Service

The majority of the study participants, 466 (82.9%) had lower awareness of selected RH services. Similarly, the large proportion of the participants, 485 (86.3%) had a unfavourable perception (Table 2).

**Table 2:** Women's perception towards selected RH services among married women in rural parts of Seden Sodo district, Southeast Ethiopia, 2023 (*N* = 562).

Statements	Strongly disagree	Disagree	Neutral	Agree	Strongly agree
Every woman of reproductive age should use MC services when they need?	9 (1.6%)	218 (39%)	36 (6.4%)	253 (45%)	46 (8%)
Every pregnant woman should use antenatal services?	0 (0%)	190 (34%)	2 (0.4%)	292 (52%)	78 (14%)
Every woman should give birth by skilled birth attendants in a health facility?	222 (40%)	0 (0%)	29 (5.2%)	241 (43%)	70 (13%)
Every woman who gave birth should use postnatal services?	1 (0.2%)	306 (54%)	21 (3.7%)	187 (33%)	47 (8%)

### 4.4 Prevalence of Decision‐Making Autonomy on RH Services Utilization

**Figure 1:**
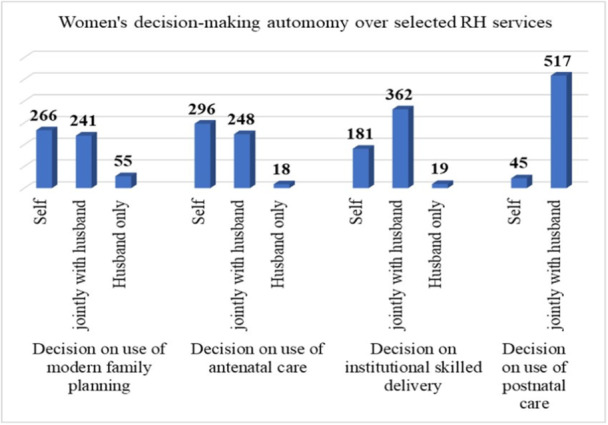
RH decision‐making autonomy of married women in rural parts of Seden Sodo district, Southeast Ethiopia, 2023 (*N* = 562).

Overall, 53.2% (95% CI: 49%‐57%) of married women have decision‐making autonomy over RH services utilization. Regarding the use of modern family planning, 47.3% of women have decision‐making autonomy for themselves. Concerning the use of ANC, about 52.7% of women usually make decisions about antenatal care by themselves. In terms of the use of institutional skilled delivery services, around 32.2% of women have decision‐making autonomy by themselves. Lastly, regarding the use of PNC, 8% of women have decision‐making autonomy for themselves (Figure 1).

### 4.5 Factors Associated With Decision‐Making Autonomy of Women

The multivariable binary logistic regression analysis showed that the odds of having autonomous decision‐making over RH services utilization is two times higher among women between the ages of 25–34 compared to women under the age of 25 (AOR: 1.93, 95% CI: 1.01–3.72). The qualitative results also reflected this age‐related dynamic, with participants stating how younger women have significant family responsibilities, restricting their ability to seek prenatal care. One participant stated, “*Since the women's primary duty was to perform housekeeping tasks, they must complete all household chores before accessing health facilities. The women began to go to the health facilities as soon as she finished her household tasks, but by the time they arrived, they had already closed*.” (A participant from Women and Children Affairs).

The odds of exercising autonomous decision‐making over RH services utilization is 53% less among women without formal education compared to those with formal education (AOR: 0.47, 95% CI: 0.30–0.76). Similarly, women whose husbands had formal education had 3.5 times higher odds of demonstrating autonomy than those with uneducated spouses (AOR: 3.51, 95% CI: 2.10–5.90). Qualitative findings also emphasized the role of education and ingrained patriarchal norms in limiting women's RH decision‐making autonomy. A health extension worker stated, *“Women were frequently expected to support their pregnancies and successfully give birth to healthy, normal kids; the ability to choose how and when to seek pregnancy and delivery care is typically not wholly owned by women.”*

Regarding occupation, women engaged as merchants had seven times greater odds of having autonomous decision‐making compared to housewives (AOR: 6.88, 95% CI: 3.12–15.14). This aligns with qualitative findings describing economic marginalization, which sustains women's dependence on men. A female healthcare provider stated, *“Many women still need permission from their partners and sometimes even their mothers‐in‐law to carry out specific tasks inside and outside the home, including asking for permission to go to a hospital or clinic to get antenatal care or give birth there.”*

Women who married at age 18 or older had six times higher odds of having RH decision‐making autonomy compared to those married earlier (AOR: 5.49, 95% CI: 3.20–9.43). Qualitative findings supported this, as participants described early marriage as fortifying male dominance and curbing women's agency. A community health worker explained, *“It's not that most women don't want to give birth at the health facility. The issue is that they have to ask their husband for permission before going to the medical facility, and they have not been allowed to leave this community.”*

Women who had parity of less than four had lower odds of decision‐making autonomy on RH services compared to those women with parity of five (AOR: 0.04, 95% CI: 0.02–0.10). When compared to their counterparts, women with favorable perceptions of RH services had a higher odd of decision‐making autonomy on RH services (AOR: 2.08, 95% CI: 1.05–4.12). Qualitative interviewees also noted that negative attitudes and mistrust toward services, often fuelled by male dominance and restricted freedom of movement, impeded women's engagement. A community health worker highlighted, *“Due to a lack of authorization to leave the house, some women choose not to visit the hospital. sometimes spouses or mothers‐in‐law won't let pregnant women leave the house.”*

The qualitative data further elucidated the socio‐cultural context restricting women's RH decision‐making autonomy: women are overburdened with household chores, limiting health facility visits; decision‐making power on RH often lies with husbands and mothers‐in‐law rather than the women themselves; women's freedom of movement is severely constrained, requiring male permission for clinic attendance or facility delivery; and economic dependency combined with male dominance traditions greatly hampers autonomous RH decisions.

One participant stated, *“The poor status of the majority of our childbearing women is, in my opinion, one of our largest concerns… many women still need permission from their partners and sometimes even their mothers‐in‐laws to carry out specific tasks inside and outside the home.” Another noted covert barriers was where husbands “claim they cannot afford to pay for the pregnant woman's travel expenses… We can only continue to raise awareness among men and community members of the value of expert care.”* (Table 3).

**Table 3:** Bivariate and Multivariable binary logistic regression analysis for determinants of women's decision‐making autonomy on selected RH services utilization in rural parts of Seden Sodo district, Southeast Ethiopia, 2023 (*N* = 562).

Variables	Decision‐making autonomy		COR (95% CI)	AOR (95% CI)
	Autonomous (%)	Non‐autonomous (%)		
Age				
18–24	89 (69)	39 (31)	1	
25–34	84 (52)	78 (48)	2.12 (1.30–3.45)	1.93 (1.01–3.72)*
35–49	126 (46)	146 (54)	2.64 (1.69–4.13)	0.66 (0.1 9–2.30)
Women's educational status				
Formal education	103 (41)	146 (59)	1	
No formal education	196 (63)	117 (37)	0.42 (0.3–0.59)	0.47 (0.30–0.76)*
Women occupation				
Housewives	269 (56)	211 (44)	1	
Merchant	30 (36)	52 (64)	2.21 (1.36–3.59)	6.88 (3.1–15.1)*
Husbands' educational status				
No formal education	242 (63)	143 (37)	1	
Formal education	57 (32)	120 (68)	3.56 (2.44–5.19)	3.51 (2.10–5.90)*
Husbands occupation				
Farmer	215 (50)	214 (50)	1	
Merchant	53 (65)	28 (35)	0.53 (0.32–0.87)	0.69 (0.36–1.31)
Daily labourer	31 (60)	21 (40)	0.68 (0.38–1.22)	0.83 (0.38–1.80)
Age at first marriage				
< 18 years	176 (70)	79 (30)	1	
≥ 18 years	123 (40)	184 (60)	3.33 (2.35–4.73)	5.49 (3.2–9.40)*
Duration since marriage				
1–10	137 (59)	96 (41)	1	
11–20	105 (58)	76 (42)	1.03 (0.70–1.53)	0.37 (0.14–0.90)
21–30	57 (39)	91 (61)	2.28 (1.50–3.47)	0.80 (0.30–2.60)
Parity (number of birth)				
≥ 5	39 (21)	148 (79)	1	
≤ 4	260 (69)	115 (31)	0.12 (0.08–0.18)	0.04 (0.02–0.10)*
Birth interval of last child				
1–2	177 (58)	129 (42)	1	
3 (more)	122 (48)	134 (52)	1.51 (1.08–2.11)	1.34 (0.76–2.35)
Awareness on RH service				
Poor awareness	270 (58)	196 (42)	1	
Good awareness	29 (30)	67 (70)	3.18 (1.98–5.11)	1.73 (0.92–3.40)
Perception towards RH services				
Unfavourable	279 (58)	206 (42)	1	
Favourable	20 (26)	57 (74)	3.86 (2.25–6.63)	2.08 (1.10–4.12)*

* Significant at *p*‐value < 0.05.

## 5 Discussion

This study aimed to assess the prevalence, determinants and barriers to RH decision‐making autonomy among married women in rural parts of Seden Sodo district, Southwest Ethiopia. The finding revealed that, over half (53.2%) of women possess autonomous RH decision‐making. This study's findings were higher than those of the studies done in rural areas of Illuababora Zone [32] and Mettu district of Ethiopia [11], that showed women's autonomous decision‐making on RH services utilization of 40.3% and 60.5% respectively. This discrepancy might be due to socio‐demographic differences. Moreover, subtle differences in the number and phrasing of decision‐making autonomy questions, cultural context, and cut‐off definitions may account for variations.

This study showed that, the odds of having autonomous decision‐making over RH services utilization is two times higher among women between the ages of 25–34 compared to women under the age of 25. This finding was consistent across multiple studies conducted in different regions [17, 33, 34, 35]. This could be because women's positions in society are socially constructed, their status varies depending on their age, and their self‐esteem increases with age.

The odds of decision‐making autonomy on RH services among women who had formal education were higher compared to those women with no formal education. This study result was in line with the study results done in Pawe [21] and Dupa [32] towns of Ethiopia. This is because education empowers women, providing them with increased decision‐making power in almost every context of RH service.

This study result revealed that women with educated husbands were four times more likely to be autonomous on RH services compared to those with no education, which is similar to the study finding in the South Gondar Zone, Northwest Ethiopia [36]. This might be because the more educated a husband, the more he will accept gender equality and believe in equal participation in decision‐making with his wife. This means that improving education has a significant impact on RH services.

This study results also showed that women who were merchants had seven times higher odds of being autonomous on their decision‐making compared to those who were housewives. The qualitative study results also showed that male dominance tradition and female economic marginalization were identified as factors that are associated with women's decision‐making autonomy in RH services utilization. This study result was in line with the study findings done in Mizan‐Aman, South Ethiopia [18]. As a result, women engaged in their own income‐generating are better able to fulfill their RH service needs independently than those who are housewives.

Compared to women whose first marriage occurred before the age of 18, those whose first marriage occurred after the age of 18 had six times higher odds of decision‐making autonomy for RH services. This finding is supported by a study finding from an Ethiopian demographic and health survey [37]. As a result, having the age of first marriage after 18 would help women exercise independence on RH service use and end male dominance in the family.

In this study, women with parity less than four were less likely to have autonomy as compared to those with five parities. In contrast, a study finding in Ambo townshowed that those women with parity greater than five were less likely to be autonomous as compared to those who were in < 5 parity [38]. This discrepancy may be due to the variation in the way the study was used to measure the autonomy of the woman and the sociocultural differences between the study areas.

Finally, those women who had favorable perceptions of RH services had higher odds of decision‐making autonomy on RH services compared to their counterparts. The qualitative study results also showed poor perceptions like attitude towards the services, male dominance traditions, and women's limited freedom of movement to access the services as factors that are associated with women's decision‐making power on RH services utilization. This study result was in line with a study done in rural Mexico [39].

### 5.1 Strengths and Limitations of the Study

The study's mixed‐methods design provides comprehensive insights by combining numerical data with qualitative context. However, the study may have limitations regarding generalizability, as the results might only apply to the specific population studied and may not easily extend to other rural regions. Reliance on self‐reported data may introduce bias, and the study focused primarily on married women, excluding other influential household members. Additionally, the data collection tool, though adapted from validated instruments, was not formally tested for reliability in this setting.

## 6 Conclusion

Women's reproductive health decision‐making autonomy in rural Ethiopia remains limited, influenced by age, education, occupation and perception toward the service. The qualitative study also showed that male dominance traditions, female economic marginalization, and women's limited freedom of movement to access the services were identified as factors that were associated with women's decision‐making autonomy on RH services utilization. As a result, empowering women through education, economic opportunities, and delaying early marriage is essential. Interventions should also engage men to shift patriarchal norms. All stakeholders must work together to promote women's education and economic development, as well as to address early marriage through awareness campaigns, legal reforms, and community involvement. Engaging males in discussion and fostering gender equality can also contribute to the creation of a situation in which women have equal decision‐making autonomy in issues pertaining to their RH service.

## Author Contributions

**Shimelis Gobena:** conceptualization, data curation, investigation, methodology, formal analysis, software. **Seifadin Ahmed Shallo:** conceptualization, methodology, project administration, supervision, software, validation. **Gemechu Gelan Bekele:** writing – original draft, methodology, validation, writing – review and editing, software, formal analysis, supervision. **Eden Girmaye Tefera:** conceptualization, methodology, project administration, supervision, visualization, validation. **Gizachew Abdissa Bulto:** conceptualization, investigation, writing – original draft, methodology, validation, visualization, writing – review and editing, software, formal analysis, project administration, supervision.

## Funding

The authors received no specific funding for this work.

## Conflicts of Interest

The authors declare no conflicts of interest.

## Transparency Statement

The lead author Gemechu Gelan Bekele affirms that this manuscript is an honest, accurate, and transparent account of the study being reported; that no important aspects of the study have been omitted; and that any discrepancies from the study as planned (and, if relevant, registered) have been explained.

## Supporting information


**Supplemental file 1:** Questionnaire.

## Data Availability

The authors confirm that the data supporting the findings of this study are available within the article. Additional datasets generated and/or analysed during the current study are available from the corresponding author upon reasonable request.
