# Supplementary material for: Prevalence, Determinants, and Barriers to Reproductive Health Decision‐Making Autonomy Among Married Women in Rural Parts of Seden Sodo District, Southwest Ethiopia: A Mixed‐Methods Study
Source: Health Sci Rep. 2026 Jan 26;9(2):e71791. doi: 10.1002/hsr2.71791 (PMC12834692; doi:10.1002/hsr2.71791)
Supplement: Supplementary file 1 — Supplemental file 1: Questionnaire. [file HSR2-9-e71791-s001.docx]

## Questionnaire

This questionnaire is designed to assess decision making power over selected reproductive health services utilization in Seden Sodo district, Oromia, Ethiopia, 2022.

Code Number ______________

Name of data collector________________________________

**Part I: Socio-Demographic Questions**

**Instruction:** Mark circle on exact response of categories and provide exact number with no category of option.

| **S. No** | **Questions** | **Response of categories** | **Skip pattern** |
| --- | --- | --- | --- |
| 101 | How old are you? | Year_____________ |  |
| 102 | What is your religion? | 1. Orthodox 2. Muslim 3. Protestant 4. Wakefeta 5. Others (Specify)   2. Female |  |
| 103 | What is your educational status? | 1. No formal education  2. grade 1-8(Primary)  3. High school(secondary)  4. College(above) |  |
| 104 | What is your occupation? | 1. House wife/ home activities 2. Daily laborer 3. Government employee 4. Farmer 5. Merchant 6. Others (Specify)   2. Urban |  |
| 105 | What is Your husband educational status? | 1. No formal education  2. grade 1-8(Primary)  3. High school(secondary)  4. College(above |  |
| 106 | What is Your husband occupation ? | 1. Daily laborer 2. Government employee 3. Farmer 4. Merchant 5. Others (Specify) |  |

**Part II. Reproductive health characteristics of study participants**

Instruction: Write possible answer on answer column provided below.

| S. N | Question to be asked | Response category | Skip pattern |
| --- | --- | --- | --- |
| 201 | How old are you when you get married? | In years_________ |  |
| 202 | How many years have you been married? | In years_________ |  |
| 203 | How many times you given birth? | In number__________ |  |
| 204 | How many years later did you have your last child from older child? | In Years_______ |  |

**Part III. Knowledge and perception of selected reproductive health services**

**Instruction:** Mark circle on exact response of categories

| **S. No** | **Knowledge questions** | **Response category** | **Skip pattern** |
| --- | --- | --- | --- |
| 301 | Have you ever heard any information about modern contraceptive services? | 1. Yes 2. No |  |
| 302 | Is there any modern contraceptive services in your area? | 1. Yes 2. No 3. I don’t know |  |
| 303 | From the following modern contraceptive methods which do you know? | 1. Oral pills 2. Injection 3. Implants 4. IUCD |  |
| 304 | Have you ever heard any information about antenatal services? | 1. Yes 2. No |  |
| 305 | Is there any antenatal services in your area? | 1. Yes 2. No 3. I don’t know |  |
| 306 | Have you ever heard any information about institutional skilled delivery services? | 1. Yes 2. No |  |
| 307 | Is there any institutional skilled delivery services in your area? | 1. Yes 2. No 3. I don’t know |  |
| 308 | Have you ever heard any information about postnatal services? | 1. Yes 2. No |  |
| 309 | Is there any post natal services in your area? | 1. Yes 2. No 3. I don’t know |  |
| **II** | **Perception questions** | **Responses** | **Skip pattern** |
| 310 | Every women of reproductive age group should use modern contraceptive services when they need? | 1. Strongly disagree 2. Dis agree 3. Neutral 4. Agree 5. Strongly agree |  |
| 311 | Every pregnant women should use ante natal services? | 1. Strongly disagree  2. Dis agree  3. Neutral  4. Agree  5. Strongly agree |  |
| 312 | Every women should gave birth by skilled birth attendants in health facility? | 1. Strongly disagree  2. Dis agree  3. Neutral  4. Agree  5. Strongly agree |  |
| 313 | Every women who gave birth should use post natal services? | 1. Strongly disagree  2. Dis agree  3. Neutral  4. Agree  5. Strongly agree |  |

**Part IV. Women’s decision-making autonomy on reproductive health services utilization**

| S. No | Questions | Response category | Skip pattern |
| --- | --- | --- | --- |
| 401 | Who in your family decides for you about use of modern family planning services? | 1.Self  2.Jointly with husband  3.Husband only |  |
| 402 | Who in your family decides for you about use of Antenatal care services? | 1.Self  2.Jointly with husband  3.Husband only |  |
| 403 | Who in your family decides for you about use of Institutional skilled delivery care services? | 1.Self  2.Jointly with husband  3.Husband only |  |
| 404 | Who in your family decides for you about use of Postnatal care services? | 1.Self  2.Jointly with husband  3.Husband only |  |

**Instruction:** Mark circle on exact response of the respondent on category column.

**Part V. socio-economic questions**

**Instruction: Encircle the right answer provided in response category**

| S. No | Presence of household possessions and HH characteristics | Response category | Skip pattern |
| --- | --- | --- | --- |
| 501 | Radio | 1. Yes 2. No |  |
| 502 | Non-mobile telephone | 1. Yes 2. No |  |
| 503 | Refrigerator | 1. Yes 2. No |  |
| 504 | Watch | 1. Yes 2. No |  |
| 505 | Table | 1. Yes 2. No |  |
| 506 | Chair | 1. Yes 2. No |  |
| 507 | Bed with cotton/sponge/spring mattress | 1. Yes 2. No |  |
| 508 | Electric mitad | 1. Yes 2. No |  |
| 509 | Kerosene lamp/pressure lamp | 1. Yes 2. No |  |
| 510 | Television | 1. Yes 2. No |  |
| 511 | Mobile phone | 1. Yes 2. No |  |
| 512 | What are your families means of transportation | 1.Bicycle  2.Animal-drawncart  3.Motorcycle/scooter  4.Car/truck/ Bajaj |  |
| 513 | Did you own agriculture land? | 1. Yes 2. No |  |
| 514 | Did you own farm animals? | 1. Yes 2. No |  |
| 515 | Did you have Electricity | 1. Yes 2. No |  |
| 516 | Flooring material Earth, sand | 1. Dung 2. Wood/bamboo 3. polished wood/ asphalt strips 4. Ceramic tiles Cement |  |
| 517 | Rooms used for sleeping | 1. One 2. Two 3. Three(more) |  |
| 518 | Place for cooking | 1. In the house  2.In a separate building  3. Outdoors  4.No food cooked in household |  |
| 519 | Cooking fuel | 1. Electricity  2. petroleum /biogas/ kerosene  3.Charcoal Wood  4.Straw/shrubs/grass  5.Agricultural crop/animal dung |  |

**Interview guide for key informant**

1. **General information of interviewee**
2. Age _____________Sex________ Educational status___________
3. Your responsibility(position)_______________________________
4. How long do you stay in the position_________________________
5. **Interview questionnaires**
6. Do you think all married women utilize reproductive health services like modern contraceptive, antenatal care, institutional health services and post natal services in your area?
7. If “yes” to question above who mostly decide to utilize the services?
8. Women( jointly with husbands)
9. Husband only
10. If “No” to question 1, what are the reason behind it?
11. What are the factors which are associated with lower decision making power of the women over reproductive health services utilization in your area? What are the solution?
